# Correction: A machine learning approach for estimating forage maize yield and quality in NW Spain

**DOI:** 10.1371/journal.pone.0337037

**Published:** 2025-11-17

**Authors:** Silverio García-Cortés, Agustín Menéndez-Díaz, María José Bande-Castro, Alfonso Carballal-Samalea, Adela Martínez-Fernández, Jose Alberto Oliveira-Prendes

There are errors in the author affiliations. The correct affiliations are as follows:

Silverio García-Cortés^1^, Agustín Menéndez-Díaz^2^, María José Bande-Castro^3^, Alfonso Carballal-Samalea^4^, Adela Martínez-Fernández^4^, Jose Alberto Oliveira-Prendes^5^

**1** Cartographic Engineering Area. University of Oviedo, Asturias, Spain, **2** Construction and Manufacturing Engineering Dept, University of Oviedo, Asturias, Spain, **3** Grassland and Crop Dept, Agricultural Research Center of Mabegondo, Galician Agency of Food Quality (Agacal), Galicia, Spain, **4** Grassland and Forage Research Program. Regional Service for Agri-food Research and Development, Asturias, Spain, **5** Plant Production Area, University of Oviedo, Asturias, Spain.

In the Acknowledgement, there is an error in the first sentence. The correct first sentence is: The authors are grateful for a grant provided by the “OECD Co-operative Research Programme” to support a research visit by the second author at the University of Florida, Gainesville, Florida, USA in 2022.
