# Correction: Correction: A machine learning approach for estimating forage maize yield and quality in NW Spain

**DOI:** 10.1371/journal.pone.0340931

**Published:** 2026-01-13

**Authors:** The PLOS One Staff

There was an error in the Acknowledgement section. The “second” author should have been listed as the “last” author. The correct Acknowledgement is as follows:

The authors are grateful for a grant provided by the OECD Co-operative Research Programme to support a research visit by the last author at the University of Florida, Gainesville, Florida, USA in 2022. The authors also gratefully acknowledge the reviewers for their insightful comments and constructive suggestions, which have greatly improved the quality of this manuscript. Finally, we acknowledge with gratitude the assistance of Ms. Christine Francis in the preparation of this paper in English.
