# Supplementary material for: A machine learning approach for estimating forage maize yield and quality in NW Spain
Source: PLoS One. 2025 Aug 12;20(8):e0326364. doi: 10.1371/journal.pone.0326364 (PMC12342283; doi:10.1371/journal.pone.0326364)
Supplement: S1 File — (DOCX) [file pone.0326364.s001.docx]

# Supporting information

Data and code can be accessed in:

# <https://doi.org/10.5281/zenodo.15470090>

Prediction web app:

<https://nwspainforagemaize.streamlit.app>

Growing Season and Radiation influence on Forage Maize yield predictions web app:

# <https://maizedrymatter.streamlit.app/>
