# Supplementary material for: A machine learning approach for estimating forage maize yield and quality in NW Spain
Source: PLoS One. 2025 Aug 12;20(8):e0326364. doi: 10.1371/journal.pone.0326364 (PMC12342283; doi:10.1371/journal.pone.0326364)
Supplement: Attachment — Submitted filename: 01_Response to ReviewersV3.docx [file pone.0326364.s003.docx]

# Response to Reviewers

Thank you very much for suggestions and corrections. We have tried to improve the document following your indications and the ones from editor. Sadly, in the main body template supplied line numbering is not working. In the following we will refer to new page numbering in the unmarked manuscript asked by the editor.

## Reviewer 1. Comment responses:

-***Page 3, L41: It is surely needed adding a new table to present calibrated genetic coefficients for each hybrid, in which, if you used calibrated genetic coefficients from other studies, they have to be presented properly.***

Thank you for your suggestion. Now in the page 4 and 5, under “**Adaptation of the CSM-CERES-Maize model**” sections, two new tables have been added. Table1 explains the CSM-CERES-Maize model parameters (genetic coefficients) and the new Table 2. Shows the values for that genetic coefficients used for each studied cultivar.

***Page 6, L3: I did not find any formulation for the Random Forest approach. All explanations are descriptive without any mathematical presentation***.

In page 12, section Random Forest Regressor we have added a new image Fig. 7 explaining the fundamental concept of the Random Forest algorithm for regression.

Also new explanations have been added and since tree decision is the key component of a random forest, also a new Table 5 in Page 12 resume the steps for the splitting node strategy (with equations) in a tree. We copied here the main text added.

*“A* ***Decision Tree*** *is also a supervised learning algorithm used for both classification and regression tasks. In the context of regression, it predicts continuous outcomes by recursively partitioning the data space into subsets based on feature values, aiming to minimize the variance within each subset. In regression, the objective of a decision tree is to split the data into subsets to minimize the loss at each node. The typical loss function minimized is the mean squared error (MSE). The reduction in MSE when splitting a node is known as "variance reduction." The goal is to find the split that maximizes this reduction.*

*where* $y_{R1}$ *is the mean of the R1 region and similarly* $y_{R2}$*. Initially, the root node receives the entire dataset of samples. As the tree grows, each split divides the data into subsets, which are then passed down to the child nodes. Splitting stops when the node contains fewer than a pre-specified minimum number of simples or when a node becomes "pure," the variance change is negligible for regression. The workflow for a decision tree can be summarized as:”*

*Compute the total error at the node:* $L_{\text{node}}=\sum_{i\in R} \left( y_{i}-\bar{y_{R}} \right)^{2}$

*For each feature* $x_{j}$ *and threshold t, compute:* $L_{\text{split}}=\sum_{i\in R_{1}} \left( y_{i}-\bar{y_{R_{1}}} \right)^{2}+\sum_{i\in R_{2}} \left( y_{i}-\bar{y_{R_{2}}} \right)^{2}$

*Calculate the variance reduction:* $\Delta L(j,t)=L_{node}-L_{split}$ *for all splits and thresholds.*

*Choose the optimal feature j and the threshold t that maximize* $\Delta L(j,t)$

***The captions for many tables and figures are not clear. For instance, in Table 2, what are theses results for? I mean for which station or site? It is very confusing. Or in Figure 8, are these results from CERES-Maize or from the RF model? This is a big problematic issue for the manuscript and must be fundamentally revised.***

Thanks for your comment. New captions with (we think) better and more explanations have been added through all the document. For example, check now the captions for: Tables 1,2, (these two tables are new), and expanded captions for Table 3 and 4 and the rest of figures.

We also have clarified the structure of the manuscript. The new structure is in our opinion clearer.

- Introduction
- Materials y methods
  - Experimental sites and minimum dataset in Asturias
  - Experimental sites and minimum dataset in Galicia
  - Adaptation of CSM-CERES Maize model
  - Simulation of the international variation in forage maize production
  - Machine learning
    - Exploratory Analysis (EDA)
    - Random Forest Regressor
- Results and discussion
  - Model fitting results
- Conclusions

***- In results and discussion section, add even few sentences about the gridded climate datasets and their benefits as an alternative for measured data and refer to*** [***https://doi.org/10.1007/s12145-023-01215-0***](https://urldefense.com/v3/__https:/doi.org/10.1007/s12145-023-01215-0__;!!D9dNQwwGXtA!ToXFwjjOP_YFBfafOQBa-P7pBRalDxt4NwFBGUQ1aVPxMJtjZODAs4FGMKgQpvV5ubKljno0h-mkh9qEdg$)***. It is important because some readers might be interested to conduct similar study as yours in their region but they cannot because of absence of measure weather data.***

Thanks again for this suggestion and the included link. We have a paragraph highlighting the importance of using the gridded meteorological dataset corresponding to precipitation, temperatures and solar radiation variables as reliable alternatives to directly measured data. Please check this addition in page 8.

Text added:

*“ Good quality long-term station data play a significant role in characterizing the climatic*

*conditions and in assessing their suitability for agricultural production [29]. Recent studies highlight the importance of using gridded meteorological datasets as reliable alternatives to directly measured data, especially in areas with sparse weather station coverage [30]. Examples of available meteorological databases include the following: NASA Power from the NASA Langley Research Center POWER (Prediction Of Worldwide Energy Resources) Project (https://power.larc.nasa.gov/) provides solar and meteorological data sets from NASA research for support of renewable energy, building energy efficiency and agricultural needs and CHIRPS (Climate Hazards Group InfraRed Precipitation with Station data) from the Climate Hazards Center, University of California, Santa Barbara for precipitation data (https://www.chc.ucsb.edu/data/chirps) [31]*

***I encourage the authors to spend more time for the preparation of the figures, because they can be much better. They are very simple.***

Our objective was to prepare as clear figures as possible, but we recognize that some of them were very simple figures. We have rethought the figures to achieve a more professional representation of the different variables involved. Thanks for your suggestion.

Figures numbered Fig2, Fig. 3 , Fig 4 and Fig. 5 and Fig. 6 are completely new. Fig. 2 includes a previous Exploratory analysis of the data in a visual manner and the rest till Fig. 6 are new. Fig 3 resume all the meteorological variables in only one chart.

***- Adding flowchart for the study workflow can improve the readability of the methodology.***

A new figure with a general flowchart has been included in the manuscript. Please check fig.1

New figure 1

Fig 1. General workflow. Left vertical part represents the field data sources and synthetic data creation using CSM-CERES and DSSAT. Right part shows the Machine learning modelling phase of the previous

New Figure 2:

**Fig 2. Dendrogram chart, grouping similar input variables and linear correlation values with respect to production vars. Input variables by rows and target var by columns.**

**Fig 7. Random Forest Regressor explanation chart. A dataset of m samples and j-features is the input of the algorithm. Each feature is represented by a small differently colored square. For each tree of the forest a different subset of samples and features is randomly chosen. Each decision tree predicts a production value based in his input subset. Finally, the mean value from all the predictions is taken.**

# About Code and Data availability:

Following the editor suggestion , although the data link: :<https://figshare.com/articles/dataset/Forage_maize_yield_and_quality_data_in_the_NW_of_Spain_Galicia_and_Asturias_/27044752?file=49239496>

included in the previous manuscript version is still working, in this second revised document we also have included a code document (as a Jupyter notebook in Python). Data file and code file are both available in this new link:

Data and code can be accessed in : <https://github.com/sgcortes/ForageMaize24>
