# Supplementary material for: A machine learning approach for estimating forage maize yield and quality in NW Spain
Source: PLoS One. 2025 Aug 12;20(8):e0326364. doi: 10.1371/journal.pone.0326364 (PMC12342283; doi:10.1371/journal.pone.0326364)
Supplement: Attachment — Submitted filename: V11_BorradorContestacionSegundoRefereePlos.docx [file pone.0326364.s004.docx]

**Review Comments to the Author**

**Reviewer #1:** The comments have been addressed and the revised manuscript looks better. So, it can be accepted now.

Thank you for your positive assessment. Many of your comments have helped us achieve a stronger manuscript.

**Reviewer #2:** The MS needs further revision to improve clarity, coherence, and scientific presentation.
My specific comments are:

1. In the abstract, the author mention that non-specialists can use this model. Is user interface or web-based tool developed? If so, please provide access details or describe its functionality.

Thank you for your suggestion. This is a goal we plan to pursue once the publication of this study in a research journal such as PLOS ONE is achieved. A preliminary version of a web app tool hosted on Streamlit Cloud is already public. A capture of its main interface was included in the text on page 23. The tool is available on: <https://nwspainforagemaize.streamlit.app>

2. Write keywords.

Thank you for the comment. You may not have been able to access the first page containing the title and the authors (please check the PDF document from PLOS ONE). On that page, the keywords have already been provided, which are as follows:

CSM-CERES-Maize model.

decision support system.

forage yield and quality.

LightGBM (Random Forest Regressor was removed)

silage maize.

machine learning.

3. Introduction and Discussion sections are poorly structured and lack clarity. The authors are encouraged to refer recent paper on ensemble machine learning techniques: [[https://doi.org/10.1016/j.atech.2024.100543](https://urldefense.com/v3/__https:/doi.org/10.1016/j.atech.2024.100543__;!!D9dNQwwGXtA!TFNfo7s18Uytvagc4TnXAQBQu3fsDKmLgsnspwPu1kgPAv0rR2SPUvE-2xPGK4nqdpC7f52RtCfe5iFoiw$)].

Following your suggestion we have tried to improve the agronomic context in the Introduction section.

On page 2 the following text was included:

*The CSM-CERES-Maize model [5] [6] [7] within the same DSSAT package has been widely used to support decision-making regarding the management of irrigation and fertilization, as well as the choice of maize cultivars. CERES-Maize continues to be the most widely used maize model globally and remains the basis of other maize models, including APSIM [8] and the CSM-IXIM [9].*

*In forage maize, [10] [11] initially adapted the CSM-CERES-Maize model (CSM = Cropping System Model) in the software provided by the Decision Support System for Agrotechnology Transfer (DSSAT) [12], in order to simulate the growth and development of three forage maize cultivars (SE1-200, SE2-300, and SE3-400) in three sites (Barcia, Villaviciosa and Grado) in Asturias.*

*One of the major problems in the selection of genotypes (cultivars) with high productivity in different environments (locations, years) is the genotype x environment interaction (GEI). Multi-environment trials (MET) often use the AMMI (additive main-effects and multiplicative interaction) model which is popular for analyzing MET data with fixed effect [13]. This model is a statistical tool for identifying GEI patterns and allows grouping genotypes according to response characteristics (identification of stable genotypes) and detecting trends between environments [14].*

On page 18 the following text was included:

*In Figure 6, we observe that the variables with the most significant influence on the training of models for all three target variables, consistently the Growing season and Radiation. Solar radiation constitutes the primary energy source for crop production. Cloudy, rainy periods that limit the amount of solar radiation available to a maize crop during susceptible stages of development contribute to regional differences that can have significant effects on yield [48]).* *Crop productivity (kg DM/ha) is a function of the amount of the Photosynthetically Active Radiation (PAR) absorbed or intercepted by the crop, which depends on incident PAR radiation and radiation use efficiency (RUE, units of g/MJ PAR) in the period of time in which it is grown, assuming that other factors are not limiting or conditioning (pests, diseases, water, nutrients, etc.) [49].*

*Beyond these, the influence of other variables varies depending on the specific target variable. However, their impact on the overall reduction of Root Mean Square Error (RMSE) is comparatively minor. This indicates that while secondary variables contribute to the model performance, their effect on improving prediction accuracy is limited compared to the primary factors.*

*Effects of temperature on biomass production and its components, radiation interception and RUE are twofold. First, and most important, temperature changes the duration of the period from sowing to harvest (Growing season). At high latitudes in Europe, Asia and North America, warming over recent decades has extended this period, with positive implications for crop growth and yield. Second, RUE is non-linearly related to temperature, an effect that is mediated by the effects of temperature on leaf gross photosynthesis, respiration and dry matter partitioning [50].*

*Maximum temperatures, which appear in fifth or sixth position as influential variable, were always below 30 ºC in our study. Maize plants are sensitive to heat stress (>30 ºC) and there is a strong decline in grain yield above this temperature when maintained for a long time [51].*

*The growing cycle of the FAO maize cultivars (200, 300 and 400) depends on the thermal time, i.e. the sum of temperatures that the maize accumulates each day from the day of sowing to the day of harvest (maize silage) or until the day of physiological maturity (maize grain).*

*Each maize cultivar has its own thermal time; the number of days needed to reach this thermal time (related to the Growing Season) varies every year. Several studies have quantified the impact of climate change, in particular the increase in temperatures on maize cultivation in Spain [52] [53]. The findings of these studies show that the increase in temperature causes a decrease in yield, even under non-water-limiting conditions, due to the shortened growing cycle. Thus, for maize forage and a given sowing date and site in a warmer than normal summer, the time to harvest will be shorter (fewer days), while in a summer with cooler than normal temperatures, the time to harvest will be longer (more days). Therefore, for the FAO cultivars (200, 300 and 400) and a given sowing date and site, a long growing cycle will be more advantageous in hot summers, and a short growing cycle will be more advantageous in cooler summers.*

4. In the Materials and Methods section, please specify the programming language, along with the libraries or packages used for data processing, modeling, and analysis.

At the bottom of page 9 the following text was included:

*Python has been used as the programming language within Jupyter Notebook enviromnment. The Python packages used include: numpy, pandas, for basic programming and sklearn, lightgbm, xgboost, optuna, shap for machine learning processes. Other auxiliary packages: joblib, matplotlib, streamlit, and plotly, were used for graphics and file outputs. The code is available at: https://doi.org/10.5281/zenodo.15470090*

5. Your study spans different environments and sowing dates, but no explicit interaction terms for genotype × environment × management are analyzed. Did you examine whether cultivar response varies significantly by location or sowing time in the ML outputs?

Thanks for your comment. We have performed a further analysis of a three-way ANOVA to cover this remark.

On page 8 the following section was included:

*2.5 Genotype × environment × management interaction*

*A three-way ANOVA was conducted to determine the effects of Cultivar, Site and Sowing date on dry matter yield. The three independent variables or factors (Cultivar, Site and Sowing date) were considered fixed factors.*

*To find out information about the three-way Site x Cultivar x Sowing date interaction (e.g., whether the three-way interaction effect is statistically significant), we need to consult the “Site x Cultivar x Sowing date" row in the Table 5.*

*Table 5. Summary of Three-Way Analysis of Variance for Site, Cultivar and Sowing date factors on dry matter yield (kg DM/ha)*

*There was no statistically significant three-way interaction between Cultivar, Site and Sowing date, F(24, 1386) = 1.287, p ≥ 0.05, but all the two-way interactions were significant.*

*There was a statistically significant two-way interaction effect between Cultivar and Site, on dry matter yield of the maize cultivars, F(12, 1386) = 5.288, p < 0.001. This indicates that cultivars were affected differently by the Sites. There was also a statistically significant two-way* *interaction effect between Site and Sowing date, F(12, 1386) = 5.775, p < 0.001 and Cultivar and Sowing date, F(4, 1386) = 4.616, p < 0.001 on dry matter yield.*

*We may follow up and interpret the two-way interactions but not the main effects due to the statistical significance of the two-way interactions. Usually when we have a significant two-way interaction (e.g. Site x Cultivar), it is the effect of this interaction that is of interest, and the main effects (e.g. Site and Cultivar) are less of interest, because, in this case, we know that the effect of Cultivar changes across levels of Site. There was a statistically significant simple main effect of Site, F(6, 1386) = 201.6, p < 0.001, Cultivar, F(2, 1386) = 447.9, p < 0.001, and Sowing date, F(2, 1386) = 617.9, p < 0.001, on Dry matter yield.*

*The graphical analysis (not presented here) showed non-crossover interactions [27] indicating that the difference in performance (kg DM/ha) of the Cultivars is not similar across the other factors (Sites or Sowing dates) but it does not change the order (ranking) of the one that produces more and the one that produces less according to the Sites or Sowing dates.*

More info is reflected here. We have not included these figures in the text but are presented here to complete the study.

Graphical analysis of double interactions not included in the text, is presented below:

We may follow up and interpret the two-way interactions but not the main effects due to the statistical significance of the two-way interactions. Usually when we have a significant two-way interaction (e.g. Site x Cultivar), it is the effect of this interaction that is of interest, and the main effects (e.g. Site and Cultivar) are less of interest, because, in this case, we know that the effect of Cultivar changes across levels of Site.

There was a statistically significant simple main effect of Site, F(6, 1386) = 201.6, p < 0.001, Cultivar, F(2, 1386) = 447.9, p < 0.001, and Sowing date, F(2, 1386) = 617.9, p < 0.001, on Dry matter yield.

We plot the Means of Dry matter yield (kg DM/ha) as a function of the interactions of Cultivar and Site. We repeat the procedure for the interaction Site and Sowing date, and the interaction Cultivar and Sowing date.

Figure ? : Means (means of three Sowing dates) of Dry matter yield (kg DM/ha) as a function of the interaction between Cultivar and Site.

 Figure ??: Means (means of three Cultivars) of Dry matter yield (kg DM/ha) as a function of the interaction between Site and Sowing date (day of the year = doy).

Figure ???: Means (means of seven sites) of Dry matter yield (kg DM/ha) as a function of the interaction between Cultivar and Sowing date (day of the year = doy).

The three figures showed non-crossover interactions (Allard and Bradshaw, 1964) indicating that the difference in performance (kg DM/ha) of the Cultivars is not similar across the other factors (Sites or Sowing dates) but it does not change the order (ranking) of the one that produces more and the one that produces less according to the Sites or Sowing dates.

In Figure ?, the latest cultivar 400 produces more than the other cultivars in the seven sites with different yields according to the site, with the Villaviciosa site having higher yields.

In Figure ??, the earliest sowing date 133 (in days of the year) is the most productive across all sites with different yields according to the site,

In Figure ???, the latest cultivar 400 produces more than the other cultivars on the three sowing dates with different yields according to the sowing date.

6. Did you directly use DSSAT output variables (like anthesis date, total biomass, nitrogen uptake) as predictors in the ML model, or were only weather, soil, and management variables used? Please clarify the feature engineering process.

Only the variables in the original excel file were used. These variables were: ['Site', 'Cultivar', 'Elevation (m)', 'Radiation (Mj/m^2^day)', 'Precipitation (mm)', 'Tmax (ºC)', 'Tmin (ºC)', 'WHC (mm)', 'C (%)', 'pH',' Sowing dates (Julian days)', 'Anthesis dates (Julian days)', 'Harvest date (Julian days)', 'Growing season (days)']. A detailed description of these variables included in the Asturian and Galician forage maize dataset generated with the CSM-CERES-Maize model were presented in Table 4.

Oliveira et al. (2023) initially adapted the CSM-CERES-Maize model in the software provided by the Decision Support System for Agrotechnology Transfer (DSSAT) for forage maize, to simulate the growth and development of three forage maize cultivars (SE1-200, SE2-300, and SE3-400) in three sites (Barcia, Villaviciosa and Grado) in Asturias. Moreover, DSSAT-based seasonal analysis was conducted to examine the interannual variation in forage maize productivity in combination with meteorological data available for 23 years (2000-2022) in the three sites in Asturias. Similar work was later performed with data from three cultivars (XU1-200, XU2-300, and XU3-400) in four sites (Ribadeo, Ordes, Deza and Sarria) in Galicia (Oliveira and Bande, 2024). The workflow carried out is summarized in Figure 1 of the manuscript.

7. Clarify the proportion of simulated vs. actual field data used for model training and testing?

The field data were observed during the years 2012–2013–2014 in Asturias and 2014–2015–2016 in Galicia. Out of the total 23 years of data, 6 years correspond to field observations and the rest are simulated, meaning that approximately 25% of the data are field based. This proportion of field data versus simulated data is expected to be maintained in both the training and test sets.

The above explanation was included now in Section 2.6 Machine Learning Processing (page 9)

Have they validated the ML model using independent field-observed data?

Sadly, no additional field data for the cultivars studied are available for years outside the study period (2012-2016). New cultivars should be then considered in future studies.

8. Have the authors considered incorporating uncertainty quantification for model predictions? For instance, bootstrapping or Bayesian Random Forest could help assess prediction robustness.

Following your suggestion, we have implemented the Bootstrap technique with the LightGBM model, which achieved the best results. Thank you for this suggestion, which we greatly appreciate. A new section 3.6 (page 23) has now been added about the results of this technique.

Also, the following text has been added to the manuscript (page 23)

*In Figure 9, the indices of the first 50 samples from the test set are shown on the x-axis (only the first 50 samples are plotted to improve the visibility of the lines and the confidence interval). The y-axis displays the values of dry matter yield per hectare. The blue line represents the actual values corresponding to these samples from the test set. The orange line corresponds to the mean yield predicted by the LightGBM model during the bootstrapping process with 100 trained models. The light blue shaded band represents the 95% confidence interval (2.5% and 97.5% percentiles) associated with those mean predicted values of the orange line.*

*In this case, the orange line closely follows the blue one, which suggests that the model captures the general trend of the actual test data well. Furthermore, the confidence interval band is narrow, indicating that the model’s confidence is high and that large variability in the predictions is not expected. The proportion of predictions for the total test set samples that fall within the 95% confidence band is approximately 55%. This relatively low percentage may suggest that there is still some bias in the data that the model has not been able to capture, or it could correspond to noise in the original data. This also corresponds to the value of the variance not modeled by the LightGBM training, as indicated by the R² metric of 0.86.*

9. Could the authors elaborate more on how cultivar and Tmax affect the predictions biologically and statistically?

After the work carried out based on the suggestions received during this review, another small app (to allow interactive exploration) for a basic sensitivity analysis of Dry matter prediction based on the values of Growing season and Radiation were created.

We believe it can give an approximate idea of what the upcoming web app for model predictions will look like: [https://maizedrymatter.streamlit.app/](https://maizedrymatter.streamlit.app/%20)

In addition, the following text has been added to the manuscript (pages 24 and 25):

*Growing season and Radiation were the variables that most influenced both the global reduction of the Root mean square error (RMSE) and the predictions (as obtained in the new variable permutation tests performed. We also calculated a set of predictions (30 by each Site studied) under conditions considered favorable for these two variables (i.e., Growing season and Radiation above their respective mean values), unfavorable (i.e., Growing season and Radiation below their means), and intermediate conditions when neither of the two thresholds—high or low—are simultaneously met. The remaining predictor variables were assigned their overall mean values, and for the categorical variable "Site", 30 samples were taken for each of the seven locations with different “Cultivar” values. The result can be seen in Figure 11.*

Figure 11 in page 25 of the manuscript

To better appreciate the result, we have developed a web app where the user can rotate and interact with the 3D figure, as well as show or hide the interpolating polynomial surface or change its degree. The web app can be accessed at: https://maizedrymatter.streamlit.app/.

The model shows a robust response, yielding higher outputs under favorable conditions for these two variables, and the opposite behavior under unfavorable conditions. This indicates stability in the predictions, with no signs of excessive variability or erratic outcomes.

10. The Random Forest algorithm is explained in too much depth for a typical agri-paper and repeated across sections. This technical detail could be moved to supplementary material to improve readability

Thank you for your suggestion. We understand your position and fundamentally agree with it. However, throughout the review process, we were asked to provide further clarifications on that, which led to the current level of detail. In this new revision, and following your recommendations, we have tried to avoid possible redundancies as you pointed out, and we have removed part of the more detailed explanation.

11. Author discuss more explicitly the limitations of using only simulation-based data, and how future studies could include more real-world datasets?

The main motivation of integration of simulation-based data into machine learning as an additional source for training data, is the augmentation of data for situations (in our paper number of the years) that are not sufficiently represented in the available data.

Future studies could include more real-world datasets because we are working together with experimental stations in Asturias and Galicia to use available data for new cultivars obtained every year of evaluation.

12. The manuscript uses only R², MAE, and MAPE. No RMSE, no prediction intervals, no feature permutation tests, no SHAP values.

Thank you for your comment. Following your suggestions, we have included in the text (pages 15 and 16) the available RMSE values (they were also calculated in the Jupyter notebooks but not included in the text), which were not reported in the previous version of the manuscript. The relative importance of the variables returned by the different models based on the training process has now been calculated for all models used.

We have also redone the Hyperparameter tuning, this time using optimization with Optuna (page 19) for tree-based models (LightGBM, XGBoost, Random Forest Regressor). This package follows an adaptive and efficient approach, using intelligent sampling algorithms (such as Bayesian Optimization and Tree-structured Parzen Estimator, TPE) to dynamically explore the hyperparameter space. For tree-based regression models, Optuna prioritizes promising regions of the search space and discards ineffective combinations through techniques such as pruning (e.g., MedianPruner). Unlike Grid Search, it does not evaluate all combinations, which significantly reduces computation time. Small improvements were achieved in the metrics wit Optuna technique.

The two variables that most strongly influence the predictions across all cases are Growing season and Radiation. These analyses were performed on the specified models after their hyperparameters had been optimized using a package called Optuna. In our experience, Optuna is particularly useful for complex models such as LightGBM and XGBoost, where the relationship between hyperparameters is non-linear and the search space is large. <https://optuna.org/>

As you suggested we have also performed variable permutation importance tests for the LightGBM models, to assess the influence of the predictor variables on the model predictions (page 20).

We have also studied the **SHAP values** (page 21) on the trained LightGBM models, as you recommended. We had never used this feature before and greatly appreciate your suggestion.

We have added this text in page.15 :

*SHAP values are an interpretability technique that explains how each feature contributes to a machine learning model’s prediction. Based on cooperative game theory developed by Lloyd Shapley, they assign an important value to each feature, quantifying its contribution to the prediction.*

And the real SHAP graphics for Dry Matter yield prediction in section 3.5 (pages 21 and 22).

13. Why is there no comparison with other ensemble machine learning techniques, such as Gradient Boosting Machines (e.g., XGBoost, LightGBM) or AdaBoost? Including such comparisons would help assess the relative performance and robustness of the Random Forest model used in this study?.

In the previous version of our work, we had trained SVR (Support Vector Machine for regression) and GradientBoost models, the latter being quite like XGBoost and implemented in scikit-learn. We have now also included LightGBM, following your valuable suggestion, as well as XGBoost and AdaBoost. The results are shown in Table 8 (pages 15 and 16).

The LightGBM method you suggested provides the best results, along with XGBoost. These two models, together with the Random Forest Regressor, have undergone hyperparameter optimization. The results are shown in Table 9 (page 19).

The improvements achieved through hyperparameter optimization are modest in terms of both R² and RMSE, but LightGBM remains the best-performing model.

14. What data splitting strategy was used to ensure no temporal data leakage?

Random sampling was applied across the entire time series (23 years) without any specific strategy to ensure a homogeneous distribution of the selected samples throughout the time. We assumed that, during the field trials, soil fertility was adjusted to similar levels at each location, and that the same cultivars were used consistently across all locations over time. Therefore, the only variables that may have changed over time are the meteorological ones, which are already explicitly included among the predictor variables. For this reason, we considered that random sampling across the entire time series was sufficient to ensure that no bias due to the time variable was introduced.

15. Try to add characteristics of cultivars on supplementary material.

A new table (Table 1) with the values of Sowing date, Anthesis date, Harvest date and the periods between sowing date and anthesis date and between sowing date and harvest date (Growing season) has been included in the new section 2.2 Cultivar Characteristics, of the manuscript (pages 4 and 5).

16. Share all DSSAT parameter files, calibration datasets, and pre-processed ML features (not just raw code) to allow full reproducibility of the study.

Table 3 (Page 6) presents the genetic coefficients of the forage maize cultivars studied, which are necessary for the calibration of the CSM-CERES-Maize model.
The calibration and evaluation of the CSM-CERES-Maize model for the studied forage maize cultivars was published by Oliveira et al., 2024, as referenced [23] in the manuscript submitted to PLOS ONE.

That earlier work was conducted in collaboration with the DSSAT research group at the University of Florida (Gainesville), and the coefficients were obtained during a research stay of one of the authors of this study within that group. At this time, we are unable to publish those results because the group at the University of Florida is still working on a new development version of the DSSAT CSM-CERES-Maize model, which will likely be released later.

17. The Conclusion section should be more concise and focused on the practical implications of the study, particularly from an agronomic and economic perspective. Rather than emphasizing that a model was “successfully developed” the authors should highlight how the model’s predictions could support cost-effective decision-making or improve resource use efficiency in forage maize production.

Following your suggestions, we have included the following text in the 4.0 Conclusion section (page 25):

*The Machine Learning models obtained have the potential use as a tool to evaluate production and quality of forage maize as affected by the geographical location of the field, cultivar type, sowing and harvesting dates, and assumptions regarding weather variables during the growing season.*

*This paper presents a procedure that allows the creation of these predictive models of yield and nutritional quality, which could be applied to other types of crops and locations.*

*To our knowledge, there are currently no other models for forage maize available for this geographic area. It is important to note that for cultivars other than those used in this study, it will be necessary to test and train a new Machine learning model based on the new data available.*

*These three prediction models (one LIghtGBM model for each target variable), are implemented on a public web app.*

7. PLOS authors have the option to publish the peer review history of their article ([what does this mean?](https://urldefense.com/v3/__https:/track.editorialmanager.com/CL0/https:*2F*2Fjournals.plos.org*2Fplosone*2Fs*2Feditorial-and-peer-review-process*23loc-peer-review-history/1/010f01960ddeee52-c746be37-0b03-4fc7-a0d2-2fe94a1507b0-000000/kA8oT4AT0G28hVZsMG40HQxUIfRnePgOllKRzfDBvi4=205__;JSUlJSUl!!D9dNQwwGXtA!TFNfo7s18Uytvagc4TnXAQBQu3fsDKmLgsnspwPu1kgPAv0rR2SPUvE-2xPGK4nqdpC7f52RtCc7pJpCNg$)). If published, this will include your full peer review and any attached files.
If you choose “no”, your identity will remain anonymous, but your review may still be made public.
The authors: **Yes**

**Do you want your identity to be public for this peer review?** For information about this choice, including consent withdrawal, please see our [Privacy Policy](https://urldefense.com/v3/__https:/track.editorialmanager.com/CL0/https:*2F*2Fwww.plos.org*2Fprivacy-policy/1/010f01960ddeee52-c746be37-0b03-4fc7-a0d2-2fe94a1507b0-000000/6ubdBocs9ltEDfT-Thhqx6h36KE6Yw6liCbrIy4TAEs=205__;JSUl!!D9dNQwwGXtA!TFNfo7s18Uytvagc4TnXAQBQu3fsDKmLgsnspwPu1kgPAv0rR2SPUvE-2xPGK4nqdpC7f52RtCf9XkP7lg$).

Reviewer #1: No

Reviewer #2: **Yes:** Dr Shankar Lal Jat

The Authors: **Yes**
